# Supplementary material for: ATP-dependent modulation of MgtE in Mg2+ homeostasis
Source: Nat Commun. 2017 Jul 27;8:148. doi: 10.1038/s41467-017-00082-w (PMC5529423; doi:10.1038/s41467-017-00082-w)
Supplement: Supplementary file 1 — Supplementary Information [file 41467_2017_82_MOESM1_ESM.pdf]

Title of file for HTML: Peer Review File
Description:

Title of file for HTML: Supplementary Information
Description: Supplementary Figures, Supplementary References.

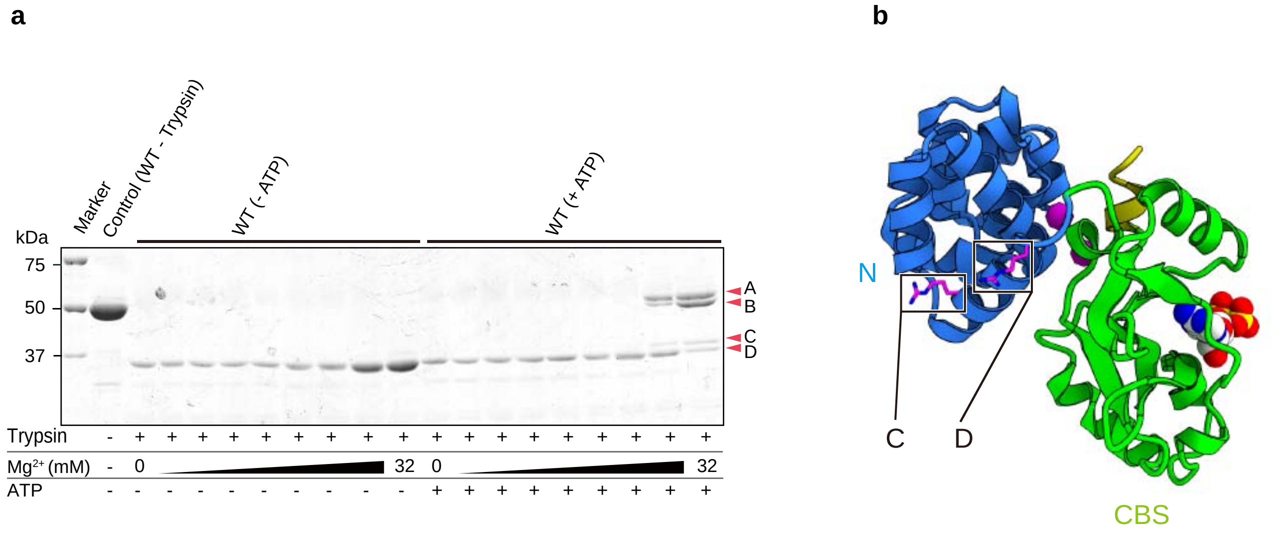

c

A B Model built

MGSSHHHHHHSSGLGVLPGGPLHMEEKLAVSLQEALQEGDTRALREVLEEIHPQDLLALWDELKGEHRYVVLTLLPKAKA

His-tag N domain

C D

AEVLSHLSPEEQAEYLKTLPPWRLREILEELSLDDLADALQAVRKEDPAYFQRLKDLLDPRTRAEVEALARYEEDEAGGL

MTPEYVAVREGMTVEEVLRFLRRAAPDAETIYYIYVVDEKGRLKGVLSLRDLIVADPRTRVAEIMNPKVVYVRTDTDQEE

CBS domain

VARLMADYDFTVLPVVDEEGRLVGIVTVDDVLDVLEAEATEDIHKLGAVDVPDLVYSEAGPVALWLARVRWLVILILTGM

Plug helix TM domain

VTSSILQGFESVLEAVTALAFYVPVLLGTGGNTGNQSATLIIRALATRDLDLRDWRRVFLKEMGVGLLLGLTLSFLLVGK

VYWDGHPLLLPVVGVSLVLIVFFANLVGAMLPFLLRRLGVDPALVSNPLVATLSDVTGLLIYLSVARLLLEAV

Supplementary Figure 1 | Cleavage sites of limited proteolysis.

(a) Protease protection of MgtE by Mg^{2+} and nucleotides, from Figure 1b. Arrows with letters indicate the four limited proteolysis products. (b) Ribbon representation of the MgtE cytosolic domain in complex with ATP. The coloring scheme is the same as that in Figure 2. ATP and Mg^{2+} ions are shown as CPK models. The arginine residues followed by the cleavage sites C and D are shown as stick models. (c) The amino acid sequences of MgtE and the cleavage sites are shown. Red arrows with letters indicate the cleavage sites.

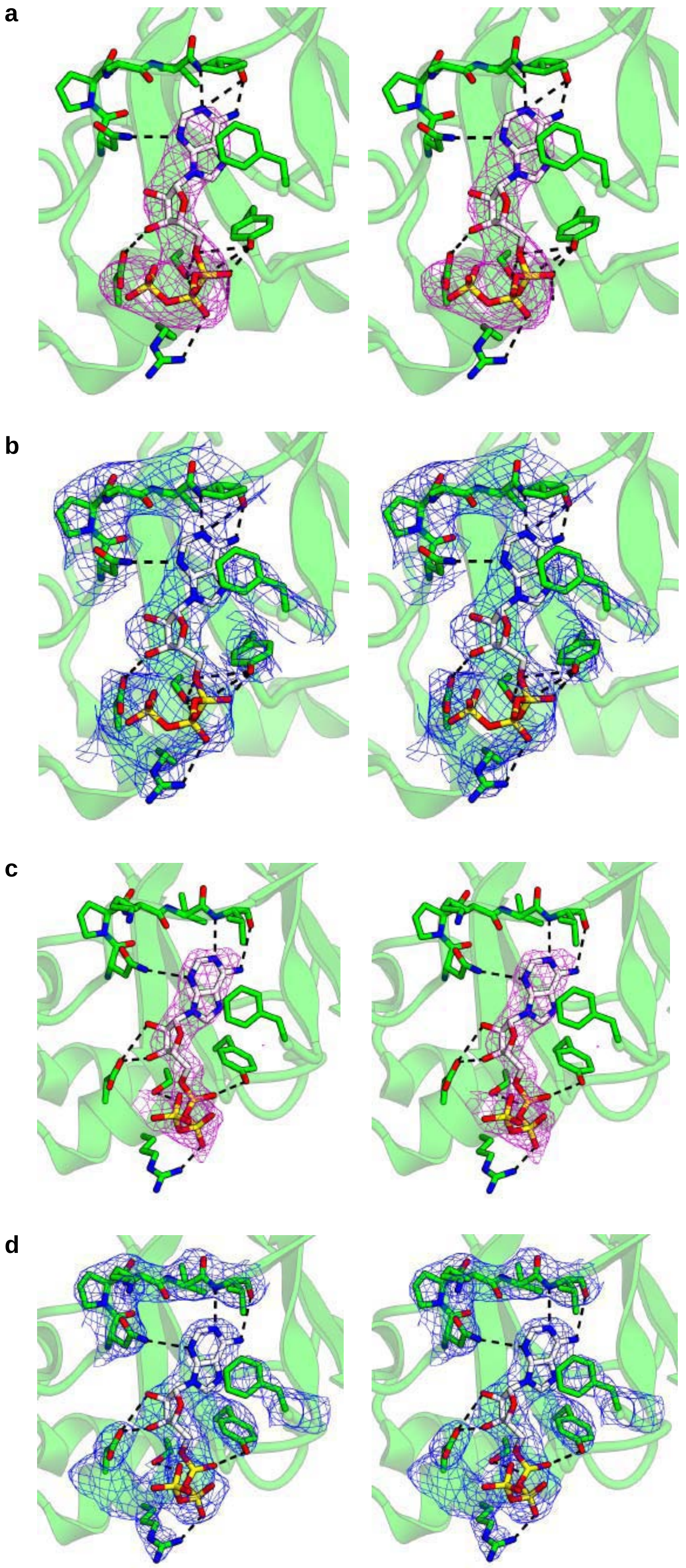

Supplementary Figure 2 | Electron density for ATP.
(a-b) Electron density maps for ATP in the full-length MgtE structure. The omit *F*o-*F*c maps (contoured at 3.0σ, green) (a) and the 2 *F*o-*F*c maps (contoured at 1.0σ, blue) (b) are shown. (c-d) Electron density maps for ATP in the cytosolic domain structure. The omit *F*o-Fc maps (contoured at 3.0σ, green) (c) and the 2 *F*o-*F*c maps (contoured at 1.0σ, blue) (d) are shown.

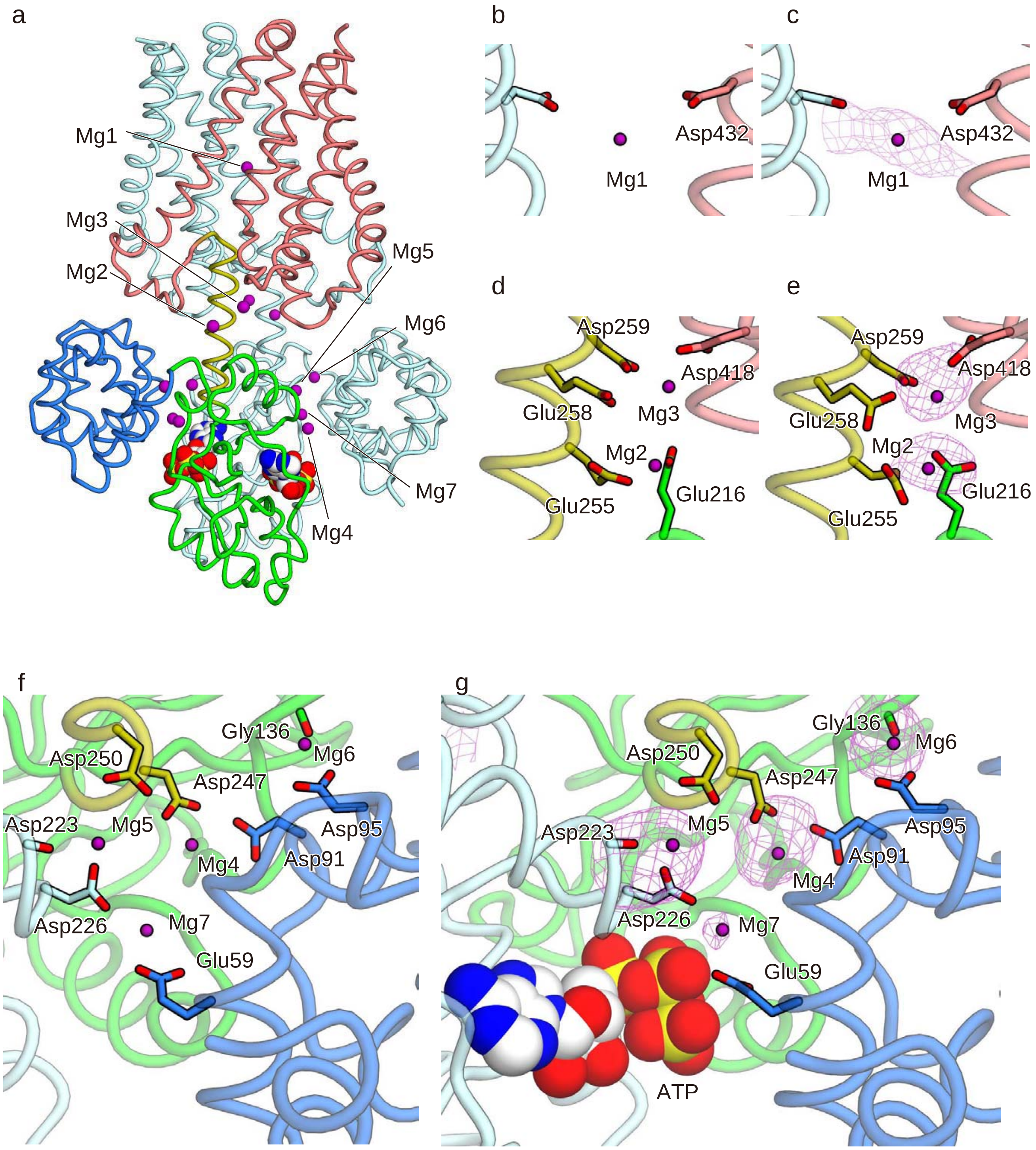

Supplementary Figure 3 | Comparison of the Mg^{2+} binding site of the full-length MgtE-ATP complex with the previously determined structure.
(a) Side view of the overall structure of the full-length MgtE-ATP complex with bound Mg^{2+}. (b-d) Close-up views of the respective Mg^{2+} [Mg1-7] binding sites in the previously determined structure of full-length MgtE. (e)–(g) Close-up views of the respective Mg^{2+} [Mg1-7] binding sites in the full-length MgtE-ATP complex, with *F*o-*F*c omit maps contoured at 2.5σ, calculated with the full-length MgtE-ATP complex excluding Mg^{2+}.

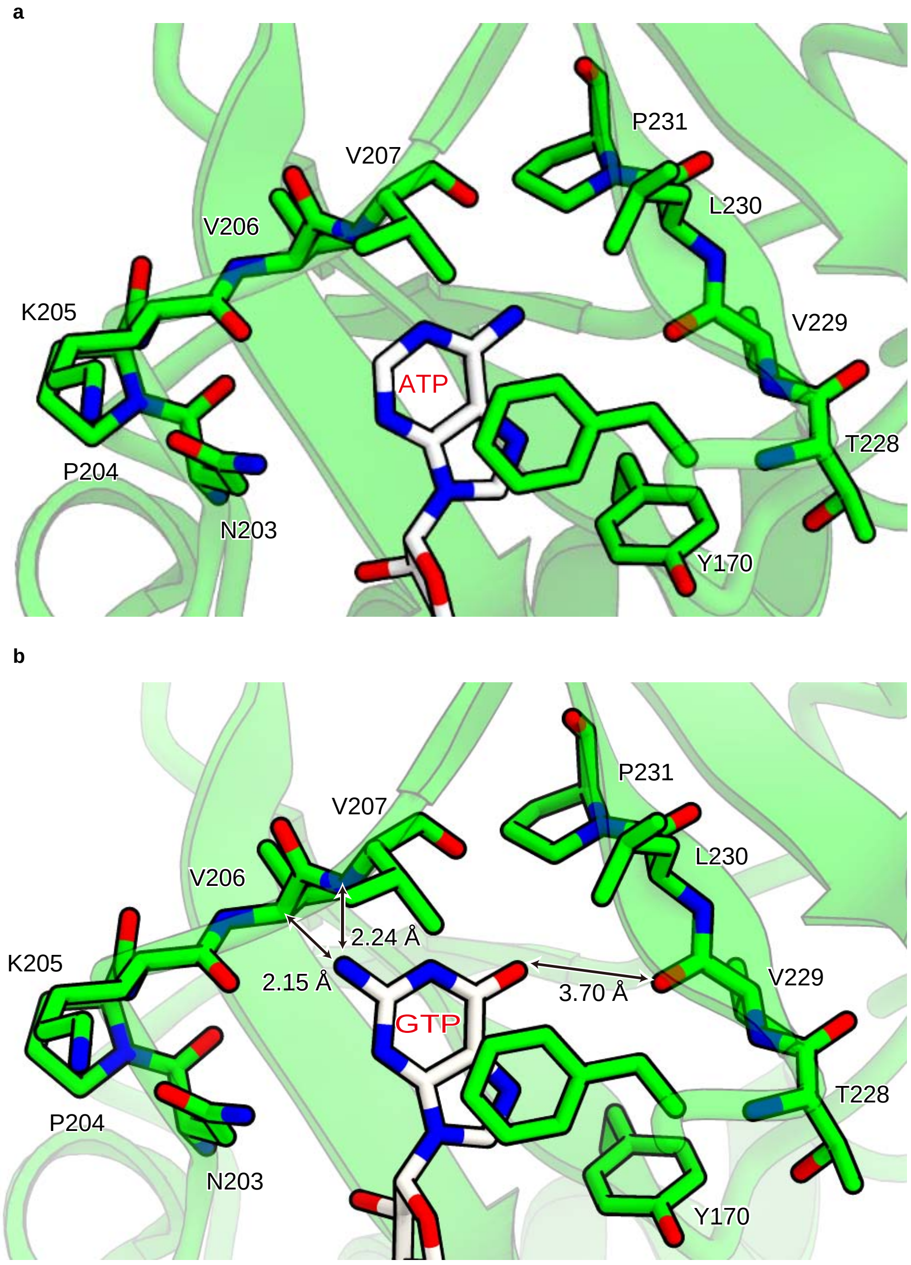

Supplementary Figure 4 | Nucleotide base specificity.

Stick representation of the ATP binding site in the MgtE cytosolic domain structure in the complex with ATP (a) and with a superimposed model of GTP (b). Distances between the N2 atom of guanine and the Cα atom of Val206, the N2 atom of guanine and the N atom of Val207, and the O6 atom of guanine and the O atom of Val229 are shown.

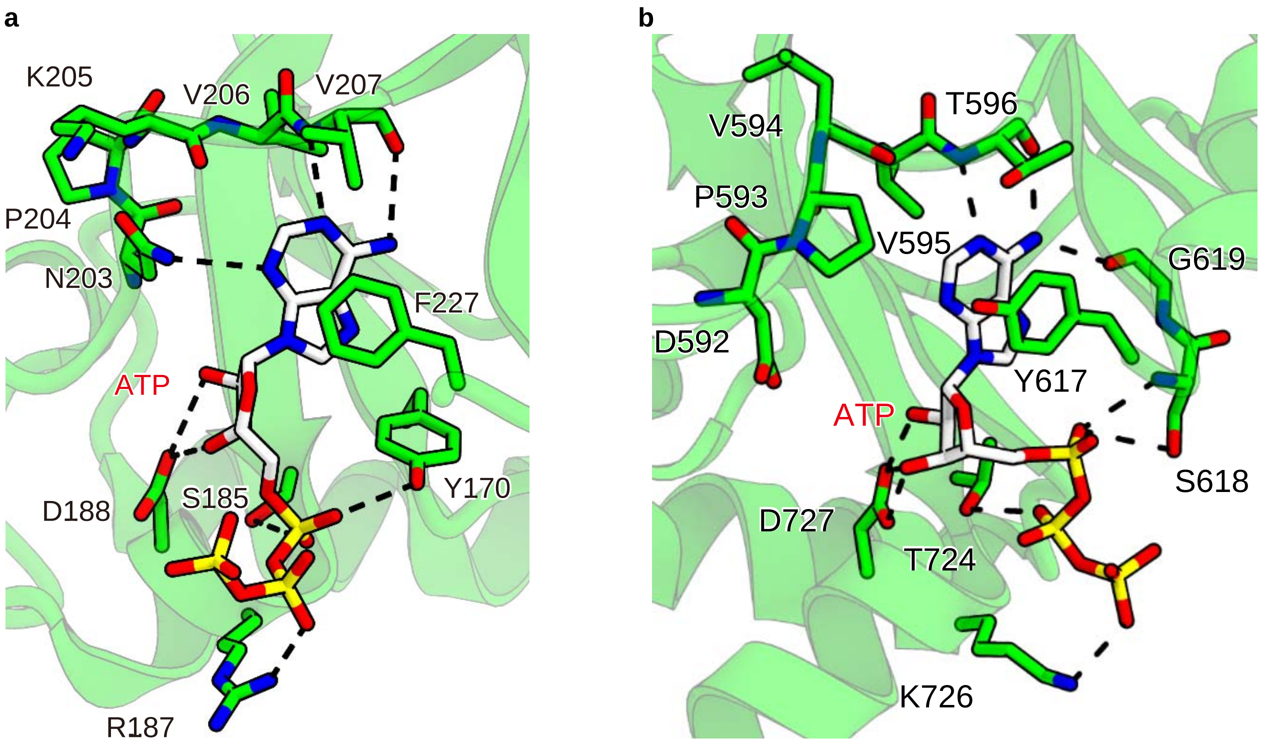

Supplementary Figure 5 | Structural comparison of CBS domains.

Structural comparison of the CBS domains of **(a)** MgtE and **(b)** human CLC-5 (PDB ID: 2J9L). The ATP binding motif (especially Arg187, Asp188, and Phe227 of MgtE) is well conserved between both domains. The Arg187, Asp188 and Phe227 of MgtE correspond to the Lys726, Asp727 and Tyr617 of human CLC-5.

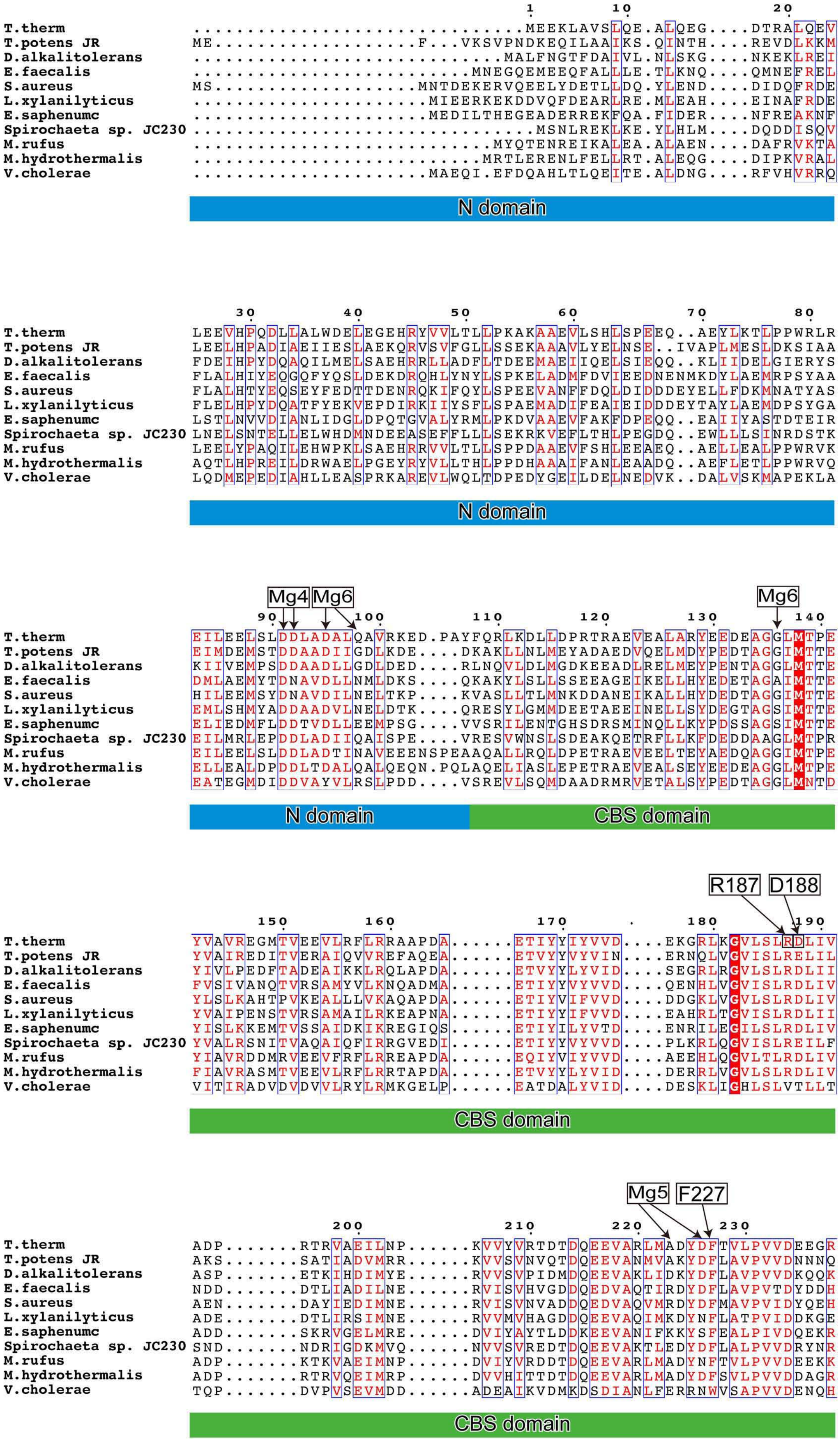

Supplementary Figure 6 | Amino acid sequence alignment of the cytosolic domain of TtMgtE with its bacterial orthologues.

The amino acid sequence alignment of TtMgtE with its orthologs was constructed with Clustal Omega[1] and ESPript 3.0[2]. Abbreviations of organisms are as follows: *T. therm., Thermus thermophilus* (Gene ID 3168925); *T. potens JR, Thermincola potens JR* (Gene ID 9149225); *D. alkalitolerans, Desulfitibacter alkalitolerans* (Gene ID 974208913); *E. faecalis, Enterococcus faecalis* (Gene ID 514904541); *S. aureus, Staphylococcus aureus* (Gene ID 581835250); *L. xylanilyticus, Lysinibacillus xylanilyticus* (Gene ID 926258026); *E. saphenum, Eubacterium saphenum* (Gene ID 492421711); *Spirochaeta sp. JC230* (Gene ID 739695846); *M. rufus, Meiothermus rufus* (Gene ID 738318087); *M. hydrothermalis, Marinithermus hydrothermalis* (Gene ID 503469645); *V. cholerae, Vibrio cholerae* (Gene ID 903058383). Strictly conserved and similar amino acid residues are highlighted with a red box and by a red letter, respectively. The amino acid residues forming Mg^{2+} binding sites and the amino acid residues involved in ATP binding are labeled on the top of the alignment. The protein domains of TtMgtE are shown below its sequence. The side chains of Asp91, Asp92, Asp95, Asn99 and Asp226, and the main chain atoms of Gly136 and Ala223 form Mg^{2+} binding sites.

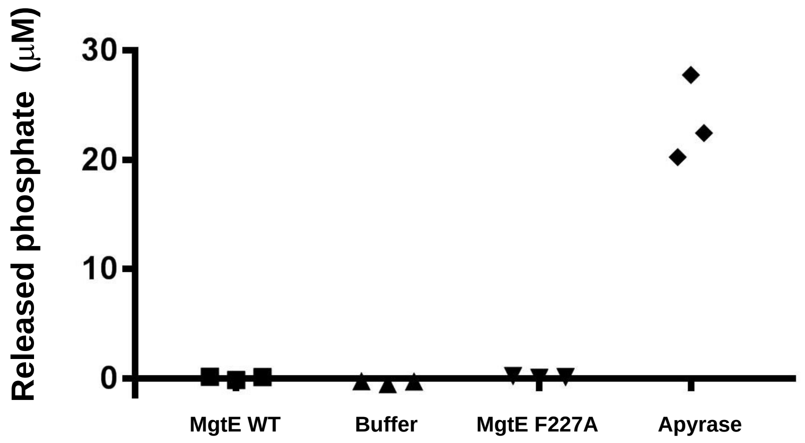

Supplementary Figure 7 | ATP hydrolysis assay of MgtE.
The ATP hydrolysis assay of MgtE. All assays were performed in triplicate. Individual measurements are plotted.

Supplementary References

1. Sievers, F. *et al.* Fast, scalable generation of high-quality protein multiple sequence alignments using Clustal Omega. *Mol. Syst. Biol.* **7,** 539 (2011).

2. Robert, X. & Gouet, P. Deciphering key features in protein structures with the new ENDscript server. *Nucleic Acids Res.* **42,** W320–4 (2014).
